# A Rewritable, Random-Access DNA-Based Storage System

**DOI:** 10.1038/srep14138

**Published:** 2015-09-18

**Authors:** S. M. Hossein Tabatabaei Yazdi, Yongbo Yuan, Jian Ma, Huimin Zhao, Olgica Milenkovic

**Affiliations:** 1 University of Illinois, Department of Electrical and Computer Engineering, Urbana, 61801, US; 2 University of Illinois, Department of Chemical and Biomolecular Engineering, Urbana, 61801, US; 3 University of Illinois, Department of Bioengineering, Urbana, 61801, US; 4 University of Illinois, Institute for Genomic Biology, Urbana, 61801, US

## Abstract

We describe the first DNA-based storage architecture that enables random access to data blocks and rewriting of information stored at arbitrary locations within the blocks. The newly developed architecture overcomes drawbacks of existing read-only methods that require decoding the whole file in order to read one data fragment. Our system is based on new constrained coding techniques and accompanying DNA editing methods that ensure data reliability, specificity and sensitivity of access, and at the same time provide exceptionally high data storage capacity. As a proof of concept, we encoded parts of the Wikipedia pages of six universities in the USA, and selected and edited parts of the text written in DNA corresponding to three of these schools. The results suggest that DNA is a versatile media suitable for both ultrahigh density archival and rewritable storage applications.

Addressing the emerging demands for massive data repositories, and building upon the rapid development of technologies for DNA synthesis and sequencing, a number of laboratories have recently outlined architectures for archival DNA-based storage^1^,^2^,^3^,^4^,^5^. The architecture in^3^ achieved a storage density of 87.5 TB/gram, while the system described in^4^ raised the density to 2.2 PB/gram. The success of the latter method may be largely attributed to three *classical coding schemes:* Huffman coding, differential coding, and single parity-check coding^4^. Huffman coding was used for data compression, while differential coding was used for eliminating homopolymers (i.e., repeated consecutive bases) in the DNA strings. Parity-checks were used to add controlled redundancy, which in conjunction with four-fold coverage allows for mitigating assembly errors.

Due to dynamic changes in biotechnological systems, none of the three coding schemes represents a suitable solution from the perspective of current DNA sequencer designs: Huffman codes are fixed-to-variable length compressors that can lead to catastrophic error propagation in the presence of sequencing noise; the same is true of differential codes. Homopolymers do not represent a significant source of errors in Illumina sequencing platforms^6^, while single parity redundancy or RS codes and differential encoding are inadequate for combating error-inducing sequence patterns such as long substrings with high GC content^6^. As a result, assembly errors are likely, and were observed during the readout process described in^4^.

An even more important issue that prohibits the practical wide-spread use of the schemes described in^3^,^4^ is that accurate partial and random access to data is impossible, as one has to reconstruct the whole text in order to read or retrieve the information encoded even in a few bases. Furthermore, all current designs support read-only storage. The first limitation represents a significant drawback, as one usually needs to accommodate access to specific data sections; the second limitation prevents the use of current DNA storage methods in architectures that call for moderate data editing, for storing frequently updated information and memorizing the history of edits. Moving from a read-only to a rewritable DNA storage system requires a major implementation paradigm shift, as:

1. Editing in the compressive domain may require rewriting almost the whole information content;
2. Rewriting is complicated by the current data DNA storage format that involves reads of length 100 bps shifted by 25 bps so as to ensure four-fold coverage of the sequence (See Fig. 1(a) for an illustration and description of the data format used in^4^). In order to rewrite one base, one needs to selectively access and modify four “consecutive” reads;
3. Addressing methods used in^3^,^4^ only allow for determining the position of a read in a file, but cannot ensure precise selection of reads of interest, as undesired cross-hybridization between the primers and parts of the information blocks may occur.

To overcome the aforementioned issues, we developed a new, random-access and rewritable DNA-based storage architecture based on DNA sequences endowed with specialized address strings that may be used for selective information access and encoding with inherent error-correction capabilities. The addresses are designed to be *mutually uncorrelated* and to satisfy the *error-control running digital sum constraint*^7^,^8^. Given the address sequences, encoding is performed by stringing together properly terminated prefixes of the addresses as dictated by the information sequence. This encoding method represents a special form of *prefix-synchronized coding*^9^. Given that the addresses are chosen to be uncorrelated and at large Hamming distance from each other, it is highly unlikely for one address to be confused with another address or with another section of the encoded blocks. Furthermore, selection of the blocks to be rewritten is made possible by the prefix encoding format, while rewriting is performed via two DNA editing techniques, the gBlock and OE-PCR (Overlap Extension PCR) methods^10^,^11^. With the latter method, rewriting is done in several steps by using short and cheap primers. The first method is more efficient, but requires synthesizing longer and hence more expensive primers. Both methods were tested on DNA encoded Wikipedia entries of size 17 KB, corresponding to six universities, where information in one, two and three blocks was rewritten in the DNA encoded domain. The rewritten blocks were selected, amplified and Sanger sequenced^12^ to verify that selection and rewriting are performed with 100% accuracy.

## Results

The main feature of our storage architecture that enables highly sensitive random access and accurate rewriting is *addressing*. The rational behind the proposed approach is that each block in a random access system must be equipped with an address that will allow for unique selection and amplification via DNA sequence primers.

Instead of storing blocks mimicking the structure and length of reads generated during high-throughput sequencing, we synthesized blocks of length 1000 bps tagged at both ends by specially designed address sequences. Adding addresses to short blocks of length 100 bps would incur a large storage overhead, while synthesizing blocks longer than 1000 bps using current technologies is prohibitively costly.

**Figure 1:**
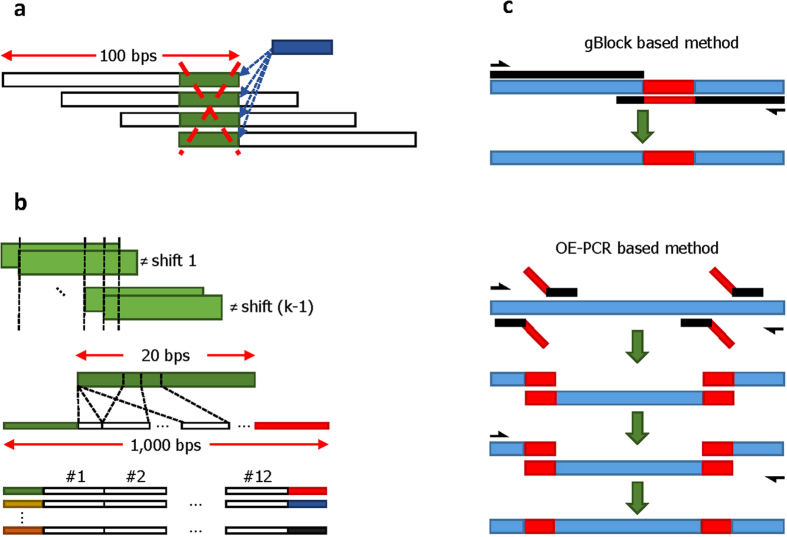
(**a**) The scheme of^4^ uses a storage format consisting of DNA strings that cover the encoded compressed text in fragments of length of 100 bps. The fragments overlap in 75 bps, thereby providing 4-fold coverage for all except the flanking end bases. This particular fragmenting procedure prevents efficient file editing: If one were to rewrite the “shaded” block, all four fragments containing this block would need to be selected and rewritten at different positions to record the new “shaded” block. (**b**) The address sequence construction process we propose which uses the notions of *autocorrelation and cross-correlation of sequences*^13^. A sequence is uncorrelated with itself if no proper prefix of the sequence is also a suffix of the same sequence. Alternatively, no shift of the sequence overlaps with the sequence itself. Similarly, two different sequences are uncorrelated if no prefix of one sequence matches a suffix of the other. Addresses are chosen to be mutually uncorrelated, and each 1000 bps block is flanked by an address of length 20 on the left and by another address of length 20 on the right (colored ends). (**c**) Content rewriting via DNA editing: the gBlock method^10^ for short rewrites, and the cost efficient OE-PCR (Overlap Extension PCR) method^11^ for sequential rewriting of longer blocks.

More precisely, each data block of length 1000 bps is flanked at both ends by two unique, yet different, address blocks of length 20 bps each. These addresses are used to provide specificity of access (see Fig. 1(b) and the Supplementary Information for details). Note that different flanking addresses simplify the process of sequence synthesis. The remaining 960 bases in a block are divided into 12 sub-blocks of length 80 bps, with each block encoding six words of the text. The “word-encoding” process may be seen as a specialized compaction scheme suitable for rewriting, and it operates as follows. First, different words in the text are counted and tabulated in a dictionary. Each word in the dictionary is converted into a binary sequence of length sufficiently long to allow for encoding of the dictionary. For our current implementation and texts of choice, described in the Supplementary Information section, this length was set to 21. Encodings of six consecutive words are subsequently grouped into binary sequences of length 126. The binary sequences are then translated into DNA blocks of length 80 bps using a new family of DNA prefix-synchronized codes described in the Methods section. Our choice for the number of jointly encoded words is governed by the goal to make rewrites as straightforward as possible and to avoid error propagation due to variable code lengths. Furthermore, as most rewrites include words, rather than individual symbols, the word encoding method represents an efficient means for content update. Details regarding the counting and grouping procedure may be found in the Supplementary Information.

For three selected access queries, the 1000 bps blocks containing the desired information were “identified” (i.e., amplified) via primers corresponding to their unique addresses, PCR amplified, Sanger sequenced, and subsequently decoded.

Two methods were used for content rewriting. If the region to be rewritten had length exceeding several hundreds, new sequences with unique primers were synthesized as this solution represents a less costly alternative to rewriting. For the case that a relatively short substring of the encoded string had to be modified, the corresponding 1000 bps block hosting the string was identified via its address, amplified and the changes were generated via DNA editing.

Both the random access and rewriting protocols were tested experimentally on two jointly stored text files. One text file, of size 4 KB, contained the history of the University of Illinois, Urbana-Champaign (UIUC) based on its Wikipedia entry retrieved on 12/15/2013. The other text file, of size 13 KB, contained the introductory Wikipedia entries of Berkeley, Harvard, MIT, Princeton, and Stanford, retrieved on 04/27/2014.

**Figure 2:**
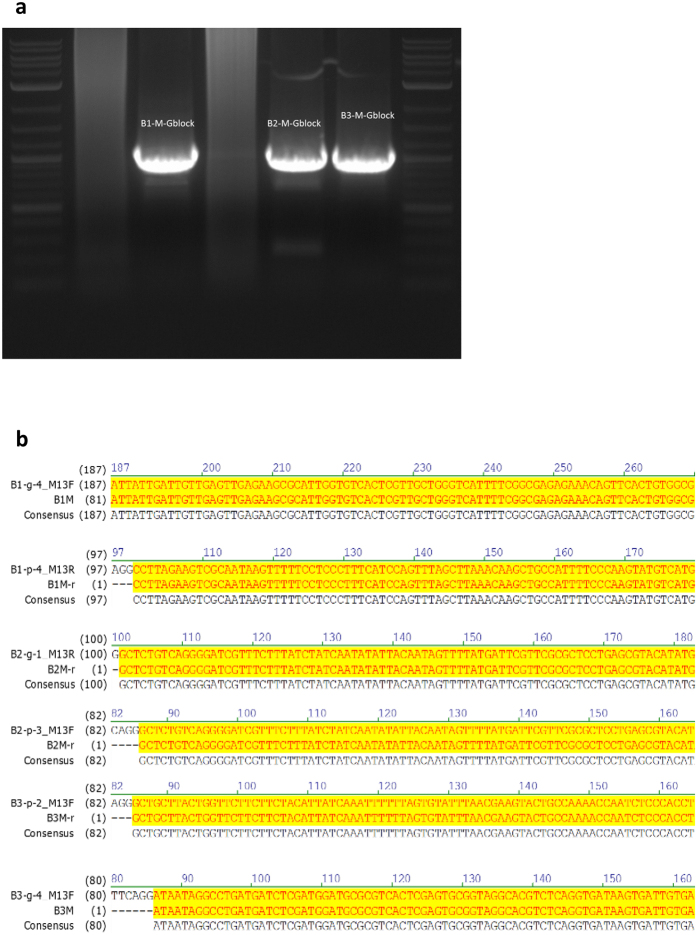
(**a**) Gel electrophoresis results for three blocks, indicating that the length of the three selected and amplified sequences is tightly concentrated around 1000 bps, and hence correct. (**b**) Output of the Sanger sequencer, where all bases shaded in yellow correspond to correct readouts. The sequencing results confirmed that the desired sequences were selected, amplified, and rewritten with 100% accuracy.

Encoded information was converted into DNA blocks of length 1000 bps synthesized by IDT (Integrated DNA Technologies), at a cost of $149 per 1000 bps (see http://www.idtdna.com/pages/products/genes/gblocks-gene-fragments). The rewriting experiments encompassed:

1. *PCR selection and amplification of one* 1000 *bps sequence and simultaneous selection and amplification of three* 1000 *bps sequences in the pool.* All 32 linear 1000 bps fragments were stored in a mixed form, and the mixture was used as a template for PCR amplification and selection. The results of amplification were verified by confirming sequence lengths of 1000 bps via gel electrophoresis (Fig. 2(a)) and by randomly sampling 3–5 sequences from the pools and Sanger sequencing them (Fig. 2(b)).
2. *Experimental content rewriting via synthesis of edits located at various positions in the* 1000 *bps blocks.* For simplicity of notation, we refer to the blocks in the pool on which we performed selection and editing as B1, B2, and B3. Two primers were synthesized for each rewrite in the blocks, for the forward and reverse direction. In addition, two different editing/mutation techniques were used, gBlock and OE-PCR. gBlocks are double-stranded genomic fragments used as primers or for the purpose of genome editing, while OE-PCR is a variant of PCR used for specific DNA sequence editing via point editing/mutations or splicing. To demonstrate the plausibility of a cost efficient method for editing, OE-PCR was implemented with general primers (≤60 bps) only. Note that for edits shorter than 40 bps, the mutation sequences were designed as overhangs in primers. Then, the three PCR products were used as templates for the final PCR reaction involving the entire 1000 bps rewrite. Figure 1(c) illustrates the described rewriting process. In addition, a summary of the experiments performed is provided in Table 1.

Given that each basepair has weight roughly equal to 650 daltons (650 × 1.67 × 10^−24^ grams), and given that 27,000 + 5000 = 32,000 bps were needed to encode a file of size 13 + 4 = 17 KB in ASCII format, we estimate a potential storage density of 4.9 × 10^20^ B/g for our scheme. This density significantly surpasses the current state-of-the-art storage density of 2.2 × 10^15^ B/g, as we avoid costly multiple coverage, use larger blocklengths and specialized word encoding schemes of large rate. A performance comparison of the three currently known DNA-based storage media is given in Table 2. We observe that the cost of sequence synthesis in our storage model is clearly significantly higher than the corresponding cost of the prototype in^4^, as blocks of length 1000 bps are still difficult to synthesize. This trend it likely to change dramatically in the near future, as within the last seven months, the cost of synthesizing 1000 bps blocks reduced almost 7-fold. Despite its high cost, our system offers exceptionally large storage density, and for the first time, enables random access and content rewriting features. Furthermore, although we used Sanger sequencing methods for our small scale experiment, for large scale storage projects Next Generation Sequencing (NGS) technologies will enable significant reductions in readout costs.

## Methods

### Address Design and Encoding

To encode information on DNA media, we employed a two-step procedure. First, we designed address sequences of short length which satisfy a number of constraints that makes them suitable for highly selective random access^13^. *Constrained coding* ensures that DNA patterns prone to sequencing errors are avoided and that DNA blocks are accurately accessed, amplified and selected without perturbing or accidentally selecting other blocks in the DNA pool. The coding constraints apply to address primer design, but also indirectly govern the properties of the fully encoded DNA information blocks. The design procedure used is semi-analytical, in so far that it combines combinatorial methods with limited computer search techniques. A unifying and highly technically charged coding approach will be reported elsewhere.

We required the address sequences to satisfy the following constraints:

- (C1) Constant GC content (close to 50%) of all their prefixes of sufficiently long length. DNA strands with 50% GC content are more stable than DNA strands with lower or higher GC content and have better coverage during sequencing. Since encoding user information is accomplished via prefix-synchronization, it is important to impose the GC content constraint on the addresses as well as their prefixes, as the latter requirement also ensures that all fragments of encoded data blocks have balanced GC content.
- (C2) Large mutual Hamming distance, as it reduces the probability of erroneous address selection. Recall that the Hamming distance between two strings of equal length equals the number of positions at which the corresponding symbols disagree. An appropriate choice for the minimum Hamming distance is equal to half of the address sequence length (10 bps in our current implementation which uses length 20 address primers). It is worth pointing out that rather than using the Hamming distance, one could use the Levenshtein (edit) distance instead, capturing the smallest number of deletions, insertions and substitutions needed to convert one string into another. Unfortunately, many address design problems become hard to analyze under this distance measure, and are hence not addressed in this manuscript.
- (C3) Uncorrelatedness of the addresses, which imposes the restriction that prefixes of one address do not appear as suffixes of the same or another address and vice versa. The motivation for this new constraint comes from the fact that addresses are used to provide unique identities for the blocks, and that their substrings should therefore not appear in “similar form” within other addresses. Here, “similarity” is assessed in terms of hybridization affinity. Furthermore, long undesired prefix-suffix matches may lead to read assembly errors in blocks during joint informational retrieval and sequencing.
- (C4) Absence of secondary (folding) structures, as such structures may cause errors in the process of PCR amplification and fragment rewriting.

Addresses satisfying constraints C1–C2 may be constructed via error-correcting codes with small running digital sum^7^ adapted for the new storage system. Properties of these codes are discussed in Section 0. The notion of *mutually uncorrelated sequences* is described in 0; it was studied in an unrelated context under the name *cross-bifix-free coding*. We also introduce a new, and more suitable version of cross-bifix-free codes termed *weakly mutually uncorrelated sequences*. Constructing addresses that simultaneously satisfy the constraints C1-C4 and determining bounds on the largest number of such sequences is prohibitively complex^14^,^15^. To mitigate this problem, we resort to a *semi-constructive* address design approach, in which balanced error-correcting codes are designed independently, and subsequently expurgated so as to identify a large set of mutually uncorrelated sequences. The resulting sequences are subsequently tested for secondary structure using *mfold* and *Vienna*^16^. In an upcoming paper, we show that the number of sequences simultaneously satisfying C1-C3 is exponentially large. We conjecture that the number of sequences satisfying all constraints, C1-C4, also grows exponentially with their length.

Given two uncorrelated sequences as flanking addresses of one block, one of the sequences is selected to encode user information via a new implementation of *prefix-synchronized encoding*^16^,^17^, described in 0. The asymptotic rate of an optimal single sequence prefix-free code is one. Hence, there is no asymptotic coding loss for avoiding prefixes of one sequence; we only observe a minor coding loss for each finite-length block. For multiple sequences of arbitrary structure, the problem of determining the optimal code rate is significantly more complicated and the rates have to be evaluated numerically, by solving systems of linear equations^17^ as described in 0 and the Supplementary Information. This system of equations leads to a particularly simple form for the generating function of mutually uncorrelated sequences.

### Balanced Codes and Running Digital Sums

One criteria for selecting block addresses is to ensure that the corresponding DNA primer sequences have prefixes with a GC content approximately equal to 50%, and that the sequences are at large pairwise Hamming distance. Due to their applications in optical storage, codes that address related issues have been studied in a different form under the name of *bounded running digital sum* (BRDS) codes^7^,^8^. A detailed overview of this coding technique may be found in^7^.

Consider a sequence *a* = *a*_0_, *a*_1_, *a*_2_, …, *a*_*l*_, …, *a*_*n*_ over the alphabet {−1, 1}. We refer to 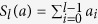 as the running digital sum (RDS) of the sequence *a* up to length *l*, *l* ≥ 0. Let 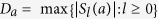 denote the largest value of the running digital sum of the sequence *a*. For some predetermined value *D* > 0, a set of sequences 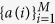 is termed a BRDS code with parameter *D* if *D*_*a*(*i*)_ ≤ *D* for all *i* = 1, …, *M*. Note that one can define non-binary BRDS codes in an equivalent manner, with the alphabet usually assumed to be symmetric, {−*q*, −*q* + 1, …, −1, 1,…, *q* − 1, *q*}, and where *q* ≥ 1. A set of DNA sequences over {A, T, G, C} may be constructed in a straightforward manner by mapping each +1 symbol into one of the bases {A, T}, and −1 into one of the bases {G, C}, or vice versa. Alternatively, one can use BRDS over an alphabet of size four directly.

To address the constraints C1–C2, one needs to construct a large set of BRDS codewords at sufficiently large Hamming distance from each other. Via the mapping described above, these codewords may be subsequently translated to DNA sequences with a GC content approximately equal to 50% for all sequence prefixes, and at the same Hamming distance as the original sequences.

Let (*n*, *C*, *d*; *D*) be the parameters of a BRDS error-correcting code, where *C* denotes the number of codewords of length *n*, *d* denotes the minimum distance of the code, while 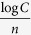 equals the code rate. For *D* = 1 and *d* = 2, the best known BRDS-code has parameters 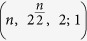, while for *D* = 2 and *d* = 1, codes with parameters 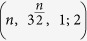 exist. For *D* = 2 and *d* = 2, the best known BRDS code has parameters 
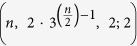
8. Note that each of these codes has an exponentially large number of codewords, among which an exponentially large number of sequences satisfy the required correlation property C3, discussed next. Codewords satisfying constraint C4 were found by expurgating the BRDS codes via computer search.

### Sequence Correlation

We describe next the notion of the autocorrelation of a sequence and describe mutually uncorrelated sequences (i.e., cross-bifix-free codes) and the new class of weakly mutually uncorrelated sequences. Mutually uncorrelated sequences, cross-bifix-free and non-overlapping codes were introduced and rediscovered many times, as witnessed by the publications^18^,^19^,^20^,^21^.

It was shown in^17^ that the autocorrelation function is the crucial mathematical concept for studying sequences avoiding forbidden strings and substrings. In the storage context, forbidden strings correspond to the addresses of the blocks in the pool. In order to accommodate the need for selective retrieval of a DNA block without accidentally selecting any undesirable blocks, we find it necessary to also introduce the notion of mutually uncorrelated sequences.

Let *X* and *Y* be two words, possibly of different lengths, over some alphabet of size *q* > 1. The correlation of *X* and *Y*, denoted by 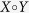, is a binary string of the same length as *X*. The *i*-th bit (from the left) of 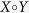 is determined by placing *Y* under *X* so that the leftmost character of *Y* is under the *i*-th character (from the left) of *X*, and checking whether the characters in the overlapping segments of *X* and *Y* are identical. If they are identical, the *i*-th bit of 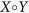 is set to 1, otherwise, it is set to 0. For example, for *X* = CATCATC and *Y* = ATCATCGG, 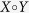 = 0100100, as depicted below.

Note that in general, 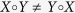, and that the two correlation vectors may be of different lengths. In the example above, we have 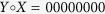. The autocorrelation of a word *X* equals 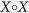.

In the example below, 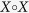 = 1001001.


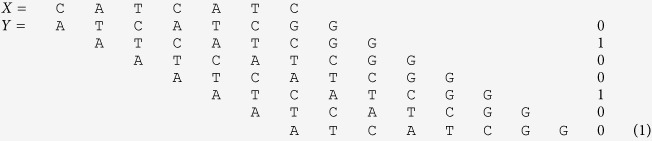


**Definition 1.** *A sequence X is self-uncorrelated if* 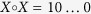. *A set of sequences* {*X*_1_, *X*_2_, …, *X*_*M*_} *is mutually uncorrelated (cross-bifix-free) if each sequence is self-uncorrelated and if all pairs of distinct sequences satisfy* 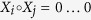 *and* 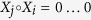.

Intuitively, correlation captures the extent to which prefixes of sequences overlap with suffixes of the same or other sequences. Furthermore, the notion of mutual uncorrelatedness may be relaxed by requiring that only sufficiently long prefixes do not match sufficiently long suffixes of other sequences. Sequences with this property, and at sufficiently large Hamming distance, eliminate undesired address cross-hybridization during selection and cross-sequence assembly errors.

We provide the following extremely simple and easy-to-prove bound on the size of the largest mutually uncorrelated set of sequences of length *n* over an alphabet of size *q* = 4. The bounds show that there exist exponentially many mutually uncorrelated sequences for any choice of *n*, and the lower bound is constructive. Furthermore, the construction used in the bound “preserves” the Hamming distance and GC content, which distinguishes it from any known results in classical coding theory.

**Theorem 2.** *Suppose that* {*X*_1_, …, *X*_*M*_} *is a set of M pairwise mutually uncorrelated sequences of length n. Let u*(*n*) *denote the largest possible value of M for a given n. Then*


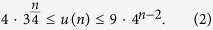


As an illustration, for *n* = 20, the *lower bound* equals 972. The proof of the theorem is give in the Supplementary Information.

It remains an open problem to determine the largest number of address sequences that jointly satisfy the constraints C1–C4. We conjecture that the number of such sequences is exponential in *n*, as the numbers of words that satisfy C1–C3 and C4^15^ are exponential. Exponentially large families of address sequences are important indicators of the scalability of the system and they also influence the rate of information encoding in DNA.

Using a casting of the address sequence design problem in terms of a simple and efficient greedy search procedure, we were able to identify 1149 sequences for length *n* = 20 that satisfy constraints C1–C4, out of which 32 pairs were used for block addressing. Another means to generate large sets of sequences satisfying the constraints is via approximate solvers for the *largest independent set problem*^22^. Examples of sequences constructed in the aforementioned manner and used in our experiments are listed in the Supplementary Information.

### Prefix-Synchronized DNA Codes

In the previous sections, we described how to construct address sequences that can serve as unique identifiers of the blocks they are associated with. We also pointed out that once such address sequences are identified, user information has to be encoded in order to *avoid* the appearance of any of the addresses, sufficiently long substrings of the addresses, or substrings similar to the addresses in the resulting DNA codeword blocks. For this purpose, we developed new prefix-synchronized encoding schemes based on ideas presented in^14^, but generalized to *accommodate multiple sequence avoidance*.

To address the problem at hand, we start by introducing comma free and prefix-synchronized codes which allow for constructing codewords that avoid address patterns. A block code 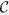 comprising a set of codewords of length *N* over an alphabet of size *q* is called *comma free* if and only if for any pair of not necessarily distinct codewords *a*_1_*a*_2_ … *a*_*N*_ and *b*_1_*b*_2_ … *b*_*N*_ in 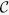, the *N* concatenations *a*_2_*a*_3_ … *a*_*N*_*b*_1_, *a*_3_*a*_4_ … *b*_1_*b*_2_, …, *a*_*N*_*a*_1_ … *b*_*N*−2_*b*_*N*−1_ are not in 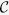
17. Comma free codes enable efficient synchronization protocols, as one is able to determine the starting positions of codewords without ambiguity. A major drawback of comma free codes is the need to implement an exhaustive search procedure over sequence sets to decide whether or not a given string of length *n* should be used as a codeword or not. This difficulty can be overcome by using a special family of comma free codes, introduced by Gilbert^9^ under the name *prefix-synchronized codes*. Prefix-synchronized codes have the property that every codeword starts with a prefix **p** = *p*_1_*p*_2_ … *p*_*n*_, which is followed by a constrained sequence 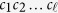. Moreover, for any codeword 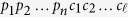 of length 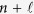, the prefix **p** *does not appear* as a substring of 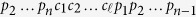. More precisely, the constrained sequences of prefix-synchronized codes avoid the pattern **p** which is used as the address. First, we point out that in our work, no consideration is given to concatenations of codewords as DNA blocks are stored unattached. Furthermore, due to the choice of mutually uncorrelated addresses at *large Hamming distance*, we can encode each information block by *avoiding only one of the address sequences*, used for that particular block. Avoidance of all other address sequences is automatically guaranteed by the lack of correlation between the sequences, as demonstrated in the proof of our encoding method.

Specifically, for a fixed set 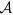 of address sequences of length *n*, we define the set 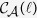 to be the set of sequences of length 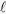 such that each sequence in 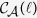 does not contain any string belonging to 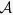. Therefore, by definition, when 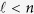, the set 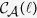 is simply the set of strings of length 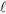. Our objective is then to design an efficient encoding algorithm (one-to-one mapping) to encode a set 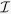 of messages into 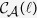. For the sake of simplicity, we let 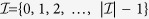.

In this scheme, we assume that 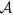 is mutually uncorrelated and all sequences in 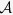 end with the same base, which we assume without loss of generality to be G. We then pick an address 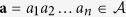 and define the following entities for 1 ≤ *i* ≤ *n*,


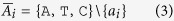



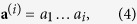


In addition, assume that the elements of 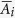 are arranged in increasing order, say using the lexicographical ordering 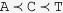. We subsequently use 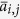 to denote the *j*-th smallest element in 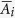, for 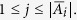 For example, if 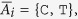 then 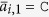 and 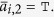


Next, we define a sequence of integers *S*_*n*,1_, *S*_*n*,2_, … that satisfies the following recursive formula


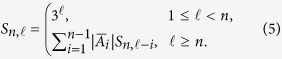


For an integer 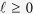 and 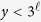, let 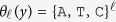 be a length-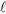 ternary representation of *y*. Conversely, for each 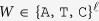, let *θ*^−1^(*W*) be the integer *y* such that 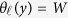. We proceed to describe how to map every integer {0, 1, 2, …, *S*_*n*,*l*_ − 1} into a sequence of length 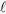 in 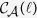 and vice versa. We denote these functions as Encode_**a**,*l*_ and Decode_**a**_, respectively.

The steps of the encoding and decoding procedures are listed in Algorithm 1


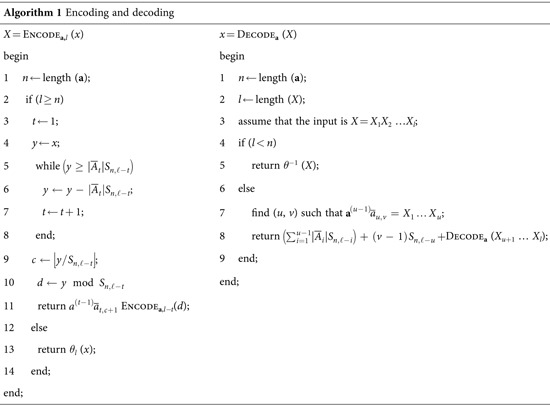


The following theorem is proved in the Supplementary Information.

**Theorem 3.** *Let* 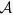 *be a set of mutually uncorrelated sequences that ends with the same base. Then for* 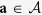, Encode_**a**,*l*_ *is an one-to-one mapping from* {0, 1, 2, …, *S*_*n*,*l*_ − 1} *to* 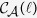
*. Moreover, for all x* ∈ {0, 1, 2, …, *S*_*n*,*l*_ − 1}, Decode_**a**_(Encode_**a**,*l*_(*x*)) = *x.*

A simple example describing the encoding and decoding procedure for the short address string **a** = AGCTG, which can easily be verified to be self-uncorrelated, is provided in the Supplementary Information.

The previously described Encode_**a**,*l*_(*x*) algorithm imposes no limitations on the length of a prefix used for encoding. This feature may lead to unwanted cross hybridization between address primers used for selection and the prefixes of addresses encoding the information. One approach to mitigate this problem is to “perturb” long prefixes in the encoded information in a controlled manner. For small-scale random access/rewriting experiments, the recommended approach is to first select all prefixes of length greater than some predefined threshold. Afterwards, the first and last quarter of the bases of these long prefixes are used unchanged while the central portion of the prefix string is cyclically shifted by half of its length. For example, for the address **a** = AGTAAGTCTCGCAGTCATCG, if the prefix **a**^(16)^ = AGTAAGTCTCGCAGTC appears as a subword, say *V*, in *X* = Encode_**a**,*l*_(*x*) then *X* is modified to *X*′ by mapping *V* to *V*^′^ = AGTAATCGGTCCAGTC. This process of shifting is illustrated below:


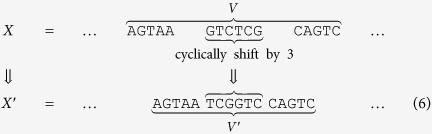


For an arbitrary choice of the addresses, this scheme may not allow for unique decoding Encode_**a**,*l*_. However, there exist simple conditions that can be checked to eliminate primers that do not allow this transform to be “unique”. Given the address primers created for our random access/rewriting experiments, we were able to uniquely map each modified prefix to its original prefix and therefore uniquely decode the readouts.

As a final remark, we would like to point out that prefix-synchronized coding also supports error-detection and limited error-correction. Error-correction is achieved by checking if each substring of the sequence represents a prefix or “shifted” prefix of the given address sequence and making proper changes when needed.

## Discussion

We described a new DNA based storage architecture that enables accurate random access and cost-efficient rewriting. The key component of our implementation is a new collection of coding schemes and the adaptation of random-access enabling codes from classical storage systems. In particular, we encoded information within blocks with unique addresses that are prohibited to appear anywhere else in the encoded information, thereby removing any undesirable cross-hybridization problems during the process of selection and amplification. We also performed four access and rewriting experiments without readout errors, as confirmed by post-selection and rewriting Sanger sequencing. The current drawback of our scheme is high cost, as synthesizing long DNA blocks is expensive. Cost considerations also limited the scope of our experiments and the size of the prototype, as we aimed to stay within a budget comparable to that used for other existing architectures. Nevertheless, the benefits of random access and other unique features of the proposed system compensate for this high cost, which we predict will decrease rapidly in the very near future.

## Additional Information

**How to cite this article**: Tabatabaei Yazdi, S. M. H. *et al.* A Rewritable, Random-Access DNA-Based Storage System. *Sci. Rep.* **5**, 14138; doi: 10.1038/srep14138 (2015).

## Supplementary Material

Supplementary Information

## Figures and Tables

**Table 1:** Selection, rewriting and sequencing results.

**Sequence identifier - Editing Method**	# **of sequence samples**	**Length of edits (bps)**	**Selection accuracy/error percentage**
B1-M-gBlock	5	20	(5/5)/0%
B1-M-PCR	5	20	(5/5)/0%
B2-M-gBlock	5	28	(5/5)/0%
B2-M-PCR	5	28	(5/5)/0%
B3-M-gBlock	5	41 + 29	(5/5)/0%
B3-M-PCR	5	41 + 29	(5/5)/0%

Each rewritten 1000 bps sequence was ligated to a linearized pCRTM-Blunt vector using the Zero Blunt PCR Cloning Kit and was transformed into *E. coli.* The *E. coli* strains with correct plasmids were sequenced at ACGT, Inc. Sequencing was performed using two universal primers: M13F_20 (in the reverse direction) and M13R (in the forward direction) to ensure that the entire block of 1000 bps is covered.

**Table 2:** Comparison of storage densities for the DNA *encoded* information expressed in B/g (bytes per gram), file size, synthesis cost, and random access features of three known DNA storage technologies.

	**Church *et al.***^3^	**Goldman *et al.***^4^	**Our scheme**
Density	0.7 × 10^15^ B/g	2.2 × 10^15 ^B/g	4.9 × 10^20^ B/g
File size	5.27 Mb	739 KB	17 KB
Cost	Not available	$12,600	$4,023
Features	Archival, no random-access	Archival, no random-access	Rewritable, random-access

Note that the density does not reflect the entropy of the information source, as the text files are encoded in ASCII format, which is a redundant representation system.

**Table 3:** 

**Algorithm 1** Encoding and decoding	
*X* = Encode_**a,***l*_ (*x*)	*x* = Decode_**a**_ (*X*)
begin	begin
1 *n* ← length (**a**);	1 *n* ← length (**a**);
2 if (*l* ≥ *n*)	2 *l* ← length (*X*);
3 *t* ← 1;	3 assume that the input is *X* = *X*_1_*X*_2_ …*X*_*l*_;
4 *y* ← *x*;	4 if (*l* < *n*)
5 while 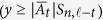	5 return *θ*^−1^ (*X*);
6 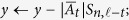	6 else
7 *t* ← *t* + 1;	7 find (*u*, *v*) such that 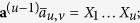
8 end;	8 return 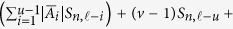 Decode_**a**_ (*X*_*u*+1_ … *X*_*l*_);
9 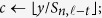	9 end;
10 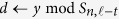	end;
11 return 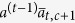 Encode_**a**,*l−t*_(*d*);	
12 else	
13 return *θ*_*l*_ (*x*);	
14 end;	
end;	
