# Supplementary material for: A Rewritable, Random-Access DNA-Based Storage System
Source: Sci Rep. 2015 Sep 18;5:14138. doi: 10.1038/srep14138 (PMC4585656; doi:10.1038/srep14138)
Supplement: Supplementary Information [file srep14138-s1.pdf]

Supplementary Information
A Rewritable, Random-Access DNA-Based Storage System

S. M. Hossein Tabatabaei Yazdi, Yongbo Yuan, Jian Ma, Huimin Zhao, Olgica Milenkovic

List of sections

1. Encoding Wikipedia Entries – A Working Example (Section 1).
2. Proofs of Theorems (Section 2).
3. Address Sequences (Section 3).
4. Example of Encoding and Decoding Procedure (Section 4).
5. Experimental Synthesis, Access and Rewrite of DNA Storage Sequences (Section 5).
6. Hybrid DNA-Based and Classical Storage (Section 6).

1 Encoding Wikipedia entries: A Working Example

In this section we describe the data format used for encoding two files of size 17 KB containing the introductory sections of Wikipedia pages of six universities: Berkeley, Harvard, MIT, Princeton, Stanford, and University of Illinois Urbana-Champaign. There were $1,933$ words in the text, out of which 842 were distinct. Note that in our context, words are elements of the text separated by a space. For example, "university" and "university." are counted as two different words, while "Urbana-Champaign" is counted as a single word. These $1,933$ words were mapped to $\lceil \frac{1933}{72} \rceil = 27$ DNA blocks of length 1000 bps, as we grouped six words into fragments, and combined 12 fragments for prefix-synchronized encoding. Table S1 provides the word counts in the files and encoding lengths (in bits) of the of the outlined procedure.

Assume that instead of using a prefix-synchronized code, we used classical ASCII encoding without compression to encode the same Wikipedia pages. The total number of characters in the text equals $12,874$, and each character is mapped to a binary string of length 7. Hence, one would need $12874 \times 7 = 90118$ bits to represent the data, which is equivalent to $\left\lceil \frac{90118}{2\times 960} \right\rceil = 47$ DNA blocks of length 1000 bps if we set aside two unique address flags for the blocks. As one can see, prefix-synchronized codes offer an almost 1.7-fold improvement in description length compared to ASCII encoding. This comes at the cost of storing a larger dictionary, as one encodes words rather than symbols of the alphabet. For the working example, one would require roughly 70-times larger dictionaries, as there are 1933 words with an average of 5.1 symbols per word. This increased in the dictionary is not a significant problem, as only one copy of the dictionary is ever needed.

2 Proofs of Theorems

Proof of Theorem 2 from the main article. The proof consists of two parts. First, we prove the upper bound on $u\,(n)$ in Lemma 1, and then proceed to prove a lower bound in Lemma 2. Recall that $u(n)$ denotes the largest possible size for a set of mutually uncorrelated words of length n.

	# symbols	# distinct symbols	# bits/distinct symbol	# bits
Characters	12874	51	6	77244
Words	1933	842	12	23196

Table S1. Comparison between character and word based encoding. Note the the number of bits per distinct symbol for the word encoding case is computed as the ceiling of the logarithm of the number of distinct symbols plus one, where the extra bit is used to prevent very small integers from being used in prefix-synchronized coding. Such integers may produce long runs of the first symbol in the address, which should be avoided. Furthermore, to ensure fixed length encoding, and hence avoid catastrophic error propagation, we doubled the number of bits used for encoding to 24.

Lemma 1. *Let $u(n)$ the largest set of distinct mutually uncorrelated sequences of length n. Then*

$$u(n) \leq 9 \cdot 4^{n-2}.$$

Proof. To prove the lemma, let us introduce some terminology. Let $d_H(\cdot,\cdot)$ stand for the Hamming distance between two words, and define the Hamming ball of radius d around a point W in $\{\mathtt{A},\mathtt{T},\mathtt{C},\mathtt{G}\}^n$ as

$$B(W,d) = \{W' \in \{\mathtt{A},\mathtt{T},\mathtt{C},\mathtt{G}\}^n : d_H(W,W') \leq d\}.$$

Furthermore, let

$$C(W,d) = \{W' \in \{\mathtt{A},\mathtt{T},\mathtt{C},\mathtt{G}\}^n : W' \in B(W,d),\ W',\ W \text{ are correlated}\}$$

denote the set of sequences correlated with W that are also at most at Hamming distance d from W.

We claim that for $n \geq d+2 \geq 4$, one has

$$|C(W,d)| \geq 2\sum_{i=0}^{d-1} \binom{n-1}{i} 3^i - \sum_{i=0}^{d-2} \binom{n-2}{i} 3^i. \tag{2.1}$$

To prove the result, assume without loss of generality that W starts with the symbol $\mathtt{A}$, i.e., $W = \mathtt{A}W_2\ldots W_n$. Next, consider two scenarios regarding the structure of $W = \mathtt{A}W_2\ldots W_n$:

- $W_n \neq \mathtt{A}$: In this case, any word W' in $B(W,d)$ that starts with W_n or ends with $\mathtt{A}$ is an element of $C(W,d)$.

  Let $S = \{W' : W' \in B(W,d),\ W' \text{ starts with } W_n\}$ and $E = \{W' : W' \in B(W,d),\ W' \text{ends with } \mathtt{A}\}$. Clearly, $|S| = |E| = \sum_{i=0}^{d-1} \binom{n-1}{i} 3^i$ and $|S \cap E| = \sum_{i=0}^{d-2} \binom{n-2}{i} 3^i$. Therefore, $|C(W,d)| \geq |S \cup E| = 2\sum_{i=0}^{d-1} \binom{n-1}{i} 3^i - \sum_{i=0}^{d-2} \binom{n-2}{i} 3^i$.

- $W_n = \mathtt{A}$: In this case, any word W' in $B(W,d)$ which starts or ends with $\mathtt{A}$ is also an element of $C(W,d)$. Using an argument similar to the one described for the previous scenario, one can show that $|C(W,d)| \geq 2\sum_{i=0}^{d} \binom{n-1}{i} 3^i - \sum_{i=0}^{d} \binom{n-2}{i} 3^i$.

  Moreover, it is straightforward to see that

$$2\sum_{i=0}^{d} \binom{n-1}{i} 3^i - \sum_{i=0}^{d} \binom{n-2}{i} 3^i > 2\sum_{i=0}^{d-1} \binom{n-1}{i} 3^i - \sum_{i=0}^{d-2} \binom{n-2}{i} 3^i.$$

For any mutually uncorrelated set $\{X_1, \ldots, X_m\}$ of size m, we have $X_i \notin C(X_1, n)$, for $2 \leq i \leq m$. This implies that

$$\{X_1, \ldots, X_m\} \subseteq \{\mathtt{A}, \mathtt{T}, \mathtt{C}, \mathtt{G}\}^n \setminus C(X_1, n).$$

At the same time, the previous claim suggests that

$$\begin{aligned} |C(X_1, n)| &\geq 2 \sum_{i=0}^{n-1} \binom{n-1}{i} 3^i - \sum_{i=0}^{n-2} \binom{n-2}{i} 3^i \\ &= 2 \cdot 4^{n-1} - 4^{n-2}. \end{aligned}$$

Therefore, $m \leq 4^n - \left(2 \cdot 4^{n-1} - 4^{n-2}\right) = 9 \cdot 4^{n-2}$, which completes the proof. □

Lemma 2. *Let $u(n)$ the largest set of distinct mutually uncorrelated sequences of length n. Then*

$$u(n) \geq 4 \cdot 3^{\frac{n}{4}}.$$

Proof. For simplicity, assume that m is even. Given a mutually uncorrelated set $\{X_1, \ldots, X_m\}$, with words of length n and over the alphabet $\{\mathtt{A}, \mathtt{T}, \mathtt{G}, \mathtt{C}\}$, partition $\{X_1, \ldots, X_m\}$ into two arbitrary sets A and B of equal size, say $A = \left\{X_1, \ldots, X_{\frac{m}{2}}\right\}$ and $B = \left\{X_{\frac{m}{2}+1}, \ldots, X_m\right\}$. We argue that $C = \{XY \mid X \in A,\, Y \in B\}$ is a mutually uncorrelated set with words of length $2n$.

- First, we show that the elements in C are self-uncorrelated: For an arbitrary element $Z \in C$, we have $Z = XY$. Since the two sequences $\{X, Y\}$ are mutually uncorrelated, one can easily verify that $Z_1^i \neq Z_{2n-i+1}^{2n}$, for $i \in \{1, \ldots, 2n-1\} \setminus \{n\}$. Moreover, since $X \neq Y$, it holds that $Z_1^n \neq Z_{n+1}^{2n}$. This establishes the claim.
- Next, we argue that any two distinct elements in C are uncorrelated: For any two distinct elements $Z = XY$ and $Z' = X'Y'$ in C, one can show that $Z_1^i \neq (Z')_{2n-i+1}^{2n}$, for $i \in \{1, \ldots, 2n-1\} \setminus \{n\}$. In addition, $X \neq Y'$ implies that $Z_1^n \neq (Z')_{n+1}^{2n}$. This completes the proof.

As a result, given a mutually uncorrelated set $\{X_1, \ldots, X_m\}$, where $X_i \in \{\mathtt{A}, \mathtt{T}, \mathtt{C}, \mathtt{G}\}^n$, one can construct another mutually uncorrelated set $\left\{Z_1, \ldots, Z_{\frac{m^2}{4}}\right\}$, where $Z_i \in \{\mathtt{A}, \mathtt{T}, \mathtt{C}, \mathtt{G}\}^{2n}$. Therefore, $u(2n) \geq \frac{u^2(n)}{4}$. Observing that for $n = 4$ it is possible to construct the following set of 12 mutually uncorrelated sequences

$$\begin{aligned} &\{\mathtt{ATGC}, \mathtt{ATAC}, \mathtt{GTAC}, \mathtt{GTGC} \\ &\mathtt{ATTC}, \mathtt{GTTC}, \mathtt{AGGC}, \mathtt{AAAC} \\ &\mathtt{GAAC}, \mathtt{GGGC}, \mathtt{ATTT}, \mathtt{GTTT}.\} \end{aligned}$$

Note that 10 sequences end with the same symbol C, while two end with the symbol T. We apply our construction by using six words that end with C as the second term in the concatenation, and using the remaining words for the first term in the concatenation. This gives an initial condition for further steps in the code construction that has parameters $n = 8$ and 36 words. The recursive concatenation procedure relying on the above base case leads $u(n) > 4 \cdot (1.31)^n$. Note that this bound is constructive, and the concatenation procedure preserves normalized minimum Hamming distances and allows you to control the GC content. □

We now turn our attention to prefix-synchronized coding, and describe a number of results relevant for our subsequent discussion.

Theorem 1 ([1]). *Given a positive integer N, chose the unique integer $n = n(N)$ so that $\beta = N2^{-n}$ satisfies*

$$\log 2 \leq \beta < 2 \log 2.$$

Then, the maximal prefix-synchronized code of length N has cardinality

$$N^{-1} 2^{N-1} \beta e^{-\beta} (1 + o(1)), \text{ as } N \to \infty,$$

for a prefix of the form $10 \ldots 0$.

Note that the above results indicate that codes avoiding one address sequence represent an exponentially large family of binary sequences. We prove a similar result for the case of 4-ary sequences that avoid a set of M mutually uncorrelated sequences. To establish the claim, we need the following definitions. Let $g(0), g(1), \ldots,$ be an integer sequence over a finite alphabet. Define the generating function of the sequence

$$G(z) = \sum_{N=0}^{\infty} g(N) z^{-N}.$$

Theorem 2. *Suppose that $\{X_1, \ldots, X_M\}$ is a set of mutually uncorrelated sequences of length n over the alphabet $\{\mathtt{A}, \mathtt{T}, \mathtt{C}, \mathtt{G}\}$. Let $f(N)$, with $f(0) = 1$, be the number of strings of length N over $\{\mathtt{A}, \mathtt{T}, \mathtt{C}, \mathtt{G}\}$ that do not contain substrings in $\{X_1, \ldots, X_M\}$. Then*

$$F(z) = \frac{z^n}{M + (z-4) z^{n-1}},$$

where $F(z)$ is the generating function of the sequence $\{f(N)\}$.

Proof. The result is a direct consequence of Theorem 4.1 of [1]. For $1 \leq i \leq M$, let $f_i(n)$ denote the number of strings of length n over $\{\mathtt{A}, \mathtt{T}, \mathtt{C}, \mathtt{G}\}$ that contain no element of $\{X_1, \ldots, X_M\}$, except for a single copy of X_i at the right-hand side of the string. Let $F_i(z)$ be the generating function of $f_i(n)$. Then, we have the following system of equations that holds for the two sets of aforementioned functions:

$$\begin{aligned} (z-4) F(z) + zF_1(z) + \ldots + zF_M(z) = z \\ F(z) - z(X_1 \circ X_1)_z F_1(z) - z(X_2 \circ X_1)_z F_2(z) - \ldots - z(X_M \circ X_1)_z F_M(z) = 0 \\ \vdots \\ F(z) - z(X_1 \circ X_M)_z F_1(z) - z(X_2 \circ X_M)_z F_2(z) - \ldots - z(X_M \circ X_M)_z F_M(z) = 0 \end{aligned} \quad (2.2)$$

By using the fact that $(X_i \circ X_i)_z = z^{n-1}$, for $1 \leq i \leq M$, and $(X_i \circ X_j)_z = 0$, for $1 \leq i \neq j \leq M$, one can show that

$$F(z) = z^n F_1(z) = \ldots = z^n F_M(z). \quad (2.3)$$

The result follows by replacing (2.2) into the first line of (2.3).

The number of sequences avoiding a set of mutually uncorrelated sequences grows roughly as ρ^n, where $\rho > 1$ is the largest pole of the generating function. □

Proof of Theorem 3 from the main article. First, we show that address $\mathbf{b} \in \mathcal{A}$ will not appear as a subword in the output $\textsc{Encode}_{\mathbf{a},\ell}$, where the output of $\textsc{Encode}_{\mathbf{a},\ell}$ equals

$$\textsc{Encode}_{\mathbf{a},\ell} = \mathbf{a}^{(t_1-1)} \bar{a}_{t_1,s_1} \ldots \mathbf{a}^{(t_r-1)} \bar{a}_{t_r,s_r} \theta_{t_0}(\cdot),$$

for some input $\theta_{t_0}(\cdot)$, and integers $1 \leq t_0, t_1, \ldots, t_r < n$. Consequently, if $\mathbf{b}$ is a substring of the output of $\textsc{Encode}_{\mathbf{a},\ell}$, then the last symbol of $\mathbf{b}$ (recall that we assumed this symbol to be $\mathtt{G}$) has to appear in one of the following three possible locations:

- The last symbol $\mathbf{b}$ appears in $\mathbf{a}^{(t_i-1)}$, for an $i \in \{1, \ldots, r\}$: In this case, there exists a suffix of $\mathbf{b}$ appearing as a prefix of $\mathbf{a}^{(t_i-1)}$. So, $\mathbf{a} \circ \mathbf{b} \neq *0\ldots0$ and this contradicts our assumption that $\mathcal{A}$ is mutually uncorrelated.
- The last symbol $\mathbf{b}$ appears in $\bar{a}_{t_i,s_i}$, for an $i \in \{1, \ldots, r\}$: This contradicts our assumption that $\bar{a}_{t_i,s_i} \neq \mathtt{G}$.
- The last symbol $\mathbf{b}$ appears in $\theta_{t_0}(\cdot)$: This contradicts our assumption that $\mathtt{G}$ does not appear in $\theta_{t_0}(\cdot) \in \{\mathtt{A}, \mathtt{T}, \mathtt{C}\}^{t_0}$.

Therefore, the string $\mathbf{b}$ does not appear as a substring in the output of $\textsc{Encode}_{\mathbf{a},\ell}$, which completes the proof of the first claim.

Next we show that for any integer $0 \leq x < S_{n,\ell}$, we have $\textsc{Decode}_{\mathbf{a}}(\textsc{Encode}_{\mathbf{a},\ell}(x)) = x$. We use induction on ℓ to establish this result. For the basis step, it is straightforward to see that

$$\begin{aligned} \textsc{Decode}_{\mathbf{a}}\left(\textsc{Encode}_{\mathbf{a},\ell}(x)\right) &= \textsc{Decode}_{\mathbf{a}}\left(\theta_{\ell}(x)\right) \\ &= \theta^{-1}\left(\theta_{\ell}(x)\right) \\ &= x, \end{aligned}$$

whenever $\ell < n$. For the inductive step, we assume that the result is true for all $\ell < r$, as well as for all $r \geq n$, and show that it is also true for $\ell = r$. Since $\ell \geq n$, one has that $\textsc{Encode}_{\mathbf{a},\ell}(x)$ first executes line 5 of the encoding algorithm. We argue that this while loop returns an integer $1 \leq t \leq n-1$. Suppose on the contrary that it does not. Then, after running the loop $n-1$ times we have $t = n-1$ and the while loop condition satisfies

$$\begin{aligned} x &\geq \sum_{i=1}^{n-1} \left|\bar{A}_i\right| S_{n,\ell-i} \\ &\overset{(a)}{=} S_{n,\ell} \end{aligned}$$

This contradicts our assumption that $0 \leq x < S_{n,\ell}$. Here, (a) follows from the definition of $S_{n,\ell}$, for $\ell \geq n$.

Hence, the encoding algorithm returns $\mathbf{a}^{(t-1)}\bar{a}_{t,c+1}\textsc{Encode}_{\mathbf{a},\ell-t}(d)$, where

$$0 \leq y = x - \sum_{i=1}^{t-1} \left|\bar{A}_i\right| S_{n,\ell-i} < \left|\bar{A}_t\right| S_{n,\ell-t}, \tag{2.4}$$

$$0 \leq c = \left\lfloor \frac{y}{S_{n,\ell-t}} \right\rfloor < \left|\bar{A}_t\right|, \tag{2.5}$$

$$0 \leq d = y \bmod S_{n,\ell-t} < S_{n,\ell-t}. \tag{2.6}$$

Next, consider $\textsc{Decode}_{\mathbf{a}}(X)$, for the input $X = \mathbf{a}^{(t-1)}\bar{a}_{t,c+1}\textsc{Encode}_{\mathbf{a},\ell-t}(d)$. Again, since $\ell \geq n$, the decoding algorithm directly executes line 7 of the decoding algorithm. We argue that $u = t$ and $v = c+1$ are the only possible outputs for the computation in step 7.

It is easy to verify that $u \leftarrow t$ and $v \leftarrow c+1$ are valid assignments, so it only remains to show that these assignments are unique. Suppose that this were not the case, and that there exists another assignment $u \leftarrow w$ and $v \leftarrow z+1$ such that $(w,z) \neq (t,c)$. We consider all possible options for w and show that all of them lead to contradictions.

- $w = t$: In this case $\mathbf{a}^{(w-1)}\bar{a}_{w,z+1} = X_1 \ldots X_{w=t} = \mathbf{a}^{(t-1)}\bar{a}_{t,c+1}$, suggesting that $\bar{a}_{t,c+1} = \bar{a}_{t,z+1}$. In addition, elements in $\bar{A}_t$ are uniquely labeled. Hence, $z = c$. This contradicts our assumption that $(w,z) \neq (t,c)$.
- $w > t$: In this case $\mathbf{a}^{(w-1)}\bar{a}_{w,z+1} = X_1 \ldots X_w$ and $\mathbf{a}^{(t-1)}\bar{a}_{t,c+1} = X_1 \ldots X_t$, implying that $\mathbf{a}^{(t-1)}\bar{a}_{t,c+1}$ is a proper prefix of $\mathbf{a}^{(w-1)}$. Therefore, $\bar{a}_{t,c+1} = a_t$. This contradicts the fact that $\bar{a}_{t,c+1} \in \{\mathtt{A},\mathtt{T},\mathtt{C}\} \setminus \{a_t\}$.
- $w < t$: This case also leads to a contradiction by invoking arguments similar to those used in the previous cases.

Hence, the "find" function uniquely identifies $(u,v) = (t,c+1)$ and line 8 of the decoding algorithm returns $\sum_{i=1}^{t-1}\left|\bar{A}_i\right| S_{n,\ell-i} + c \times S_{n,\ell-t} + \textsc{Decode}_{\mathbf{a}}\left(\textsc{Encode}_{\mathbf{a},\ell-t}(d)\right)$. Now, the proof may be completed by noticing

Designation of primer	Sequence
B1-forward	5′AATTACTAAGCGACCTTCTC3′
B1-reverse	5′ACTTATTGCGACTTCTAAGG3′
gBlock-B1-reverse	5′CTTCATAACAACTAACTGTGAC3′
B1-SU1-reverse	5′CGTGCACTCATAACCCATATTTCAAGAGCT<br>AGCTATTCCTCTCCCTTAAAAGTAAATGAC3′
B1-SD1-forward	5′GGGAGAGGAATAGCTAGCTCTTGAAATAT<br>GGGTTATGAGTGCACGATCATCACATAAC3′
B2-forward	5′AACCTAACCATCTTCCTCTC3′
B2-reverse	5′AAACGATCCCCTGACAGAGC3′
gBlock-B2-forward	5′GAAGCACAGTGTTGCTGCGTG3′
B2-SU1-reverse	5′CAGCTTGTATCCCATCTCAACCCTAATTC<br>CATAACCGTCAGCGCAGTTGACTAGTCTC3′
B2-SD1-forward	5′CTGCGCTGACGGTTATGGAATTAGGGTT<br>GAGATGGGATACAAGCTGATATGGGAAC3′
B3-forward	5′ATAATAGGCCTGATGATCTC3′
B3-reverse	5′AAGAAGAACCAGTAAGCAGC3′
B3-SU1-reverse	5′AACATCTACTCACTCTCAATCTAAGCTTGA<br>ACTGTGTACACACCATCGCTCTTGTACGCC3′
B3-SU2-forward	5′GTGTACACAGTTCAAGCTTAGATTGAGAGT<br>GAGTAGATGTTGATGCGAGGCGAAAGATGT3′
B3-SD2-reverse	5′GACTTCCCCCCTATAATCCATTAATGCTAG<br>ATCAAGCCGCATATACTATGTTGCAAATAC3′
B3-SD2-forward	5′GCGGCTTGATCTAGCATTAATGGATTA<br>TAGGGGGGAAGTCGCTGCTGGTACTCTG3′

Table S2. List of primers for rewriting (editing) the blocks B1, B2 and B3. The primers for the gBlock method are listed separately for those used with the OE-PCR method. In the latter case, the labels of DNA fragments SU and SD stand for sample upstream and sample downstream. In OE-PCR, we linked two DNA fragments or three DNA fragments into the final PCR products; when two fragments were linked, the first fragment was labeled UP (U), while the second fragment was labeled DOWN (D); when three fragments were combined, the second fragment was labeled MIDDLE (M).

the validity of the following steps

$$\begin{aligned}
\textsc{Decode}_{\mathbf{a}}\left(\textsc{Encode}_{\mathbf{a},\ell}(x)\right) &= \textsc{Decode}_{\mathbf{a}}\left(\mathbf{a}^{(t-1)}\bar{a}_{t,c+1}\textsc{Encode}_{\mathbf{a},\ell-t}(d)\right) \\
&= \sum_{i=1}^{t-1}\left|\bar{A}_i\right| S_{n,\ell-i} + c \times S_{n,\ell-t} + \textsc{Decode}_{\mathbf{a}}\left(\textsc{Encode}_{\mathbf{a},\ell-t}(d)\right) \\
&\overset{(a)}{=} (x-y) + c \times S_{n,\ell-t} + \textsc{Decode}_{\mathbf{a}}\left(\textsc{Encode}_{\mathbf{a},\ell-t}(d)\right) \\
&\overset{(a)}{=} (x-y) + (y-d) + \textsc{Decode}_{\mathbf{a}}\left(\textsc{Encode}_{\mathbf{a},\ell-t}(d)\right) \\
&\overset{(c)}{=} x
\end{aligned}$$

Here, (a) follows from (2.4), (b) follows from (2.5) and (2.6), and (c) follows from the fact that $\ell - t < r$ and $0 \leq d < S_{n,\ell-t}$. By the induction hypothesis we therefore have $\textsc{Decode}_{\mathbf{a}}\left(\textsc{Encode}_{\mathbf{a},\ell-t}(d)\right) = d$.

3 Address Sequences

Consider the following set of strings of length 20,

CGTAGTCAGCGTGTCAATCA
TGCACAGTCGAGCTATCACA
GACTGACTGATGACGACTGA
GCTATATGCGAGTCGAGTCA
GTACACTCAGCATCGACTCA

with GC content equal to 50%, i.e., 10 GC bases. The sequences are mutually uncorrelated and at Hamming distance exactly 10 from each other. The sequences do not exhibit secondary structures at room temperature, as verified by the mfold and Vienna packages. We used these addresses for a very small-scale, proof-of-concept random access/rewriting experiment of a 4 KB file.

In the large scale random access/rewriting experiment described in Section 5, we used different address sequences for the two flanking ends of the 1000 bps blocks. The sequences we synthesized include:

block 1: (AATTACTAAGCGACCTTCTC, ACTTATTGCGACTTCTAAGG)
block 2: (AACCTAACCATCTTCCTCTC, AAACGATCCCCTGACAGAGC)
block 3: (ATAATAGGCCTGATGATCTC, AAGAAGAACCAGTAAGCAGC)
block 4: (AAAACACGTGTCGCTTTCTC, AAATCGGAAATTCGTGTCGC)
block 5: (AAGTGTGTAAAGGTCGTCTC, AATTCACGGTCCGAAACACC)
block 6: (TGCTCTTTCCTCCTTGTCTC, TGTAGACGATTTGATTGGCG)
block 7: (TAAACGCCTTCAACGATCTC, ACGAGATTCATACCGGACCC)
block 8: (ATACCTATCCCTTCGATCTC, TGCAGAAGAGGAGTGTCAGC)
block 9: (TGTATGGTCTCGGATATCTC, TTTAACCCGCCCGTACAGCC)
block 10: (TTTGTACTCTACTCGCTCTC, ACAGTACTTGCCCAATTCGC)
block 11: (ACTAAGTCGCCTCATGTCTC, TAAACATTACAAGCCCCTCG)
block 12: (TGTAGCAGTCCCTTCTTCTC, AATACAACTTCTAACCACCC)
block 13: (AAAGAGTCATCCTAGTTCTC, TTAATAGTTCCCGGCAGCCC)
block 14: (ATGGACAGTGCAGTGATCTC, TTAGAACGAACCAGTATAGC)
block 15: (AAGTTTCCGGAATCCATCTC, TTGACCCATGAGCCAGCACC)
block 16: (TGCTCAAATGATGACATCTC, TGCTGAACTCTAATCTGTCC)
block 17: (AACACATGTCGGCGGGTCTC, ATACACTCATAACACCTCGG)
block 18: (TTGAAAAAACACTAGCGTCTC, ACAACTATACGTGTCGGACC)
block 19: (TATCCTGAGCACGATTTCTC, TGAACCCGTCGTGCTAATCG)
block 20: (TTACCCGCACGCATAATCTC, ATACGGGATACAATTAGGGC)
block 21: (TTTTATAGGTGCGGAGTCTC, AATACATCCCTAAAAGCCGG)
block 22: (TTACCTTACTTGTGCGTCTC, TGAGGATAGGATTAGTAAGG)
block 23: (TACGTCAGTCTAAGAATCTC, ATGTTAACACTGAGTAAGGG)
block 24: (ACTGTACCCAAGCTAGTCTC, ACATGACCTACATAACGTCC)
block 25: (TAAAAATCCGGTGGTCTCTC, AACAGAGATCAGAGCAGTGG)
block 26: (TGAAGTTGCAAAGAGATCTC, AACCCGTACTCACTATGCCG)
block 27: (TACAACACATCTGCAGTCTC, TTTGTAGATCCCAAGCATCG)

The pairs of sequences were used to flank the two ends of the data blocks. Only the addresses on the left were used for subsequent prefix-synchronized coding, and they all end with the same symbol – C.

The sequences on the left-hand side of the pairing have "interleaved" $\{\mathtt{G},\mathtt{C}\}$ and $\{\mathtt{A},\mathtt{T}\}$ bases – for example, they all start with CTCT.... This ensures a "GC balancing" property for the prefixes of the addresses.

4 Encoding and Decoding Example

In this section, we illustrate the encoding and decoding procedure for the short address string $\mathbf{a} = \mathtt{AGCTG}$, which can easily be verified to be self-uncorrelated.

More precisely, we explain how to compute a sequence of integers $S_{n,1}, S_{n,2}, \ldots, S_{n,7}$, described in the main body of the paper. As before, n denotes the length of the address string, which in this case equals five.

One has

$$(S_{n,1}, S_{n,2}, \ldots, S_{n,7}) = (3, 9, 27, 81, 267, 849, 2715)\,.$$

The algorithm $\textsc{Encode}_{\mathbf{a},8}(550)$ produces:

$550 = 0 \times S_{5,7} + 550$
$\Rightarrow \textsc{Encode}_{\mathbf{a},8}(550) = \underline{\mathtt{C}}\textsc{Encode}_{\mathbf{a},7}(550)$
$550 = 0 \times S_{5,6} + 550$
$\Rightarrow \textsc{Encode}_{\mathbf{a},7}(550) = \underline{\mathtt{C}}\textsc{Encode}_{\mathbf{a},6}(550)$
$550 = 2 \times S_{5,5} + 0 \times S_{5,4} + 16$
$\Rightarrow \textsc{Encode}_{\mathbf{a},6}(550) = \underline{\mathtt{AA}}\textsc{Encode}_{\mathbf{a},4}(16),$
$16 = 0 \times 3^3 + 1 \times 3^2 + 2 \times 3^1 + 1 \times 3^0$
$\Rightarrow \textsc{Encode}_{\mathbf{a},4}(16) = \underline{\mathtt{ATCT}},$
$\Rightarrow \textsc{Encode}_{\mathbf{a},8}(550) = \underline{\mathtt{CCAAATCT}}$

When running $\textsc{Decode}_{\mathbf{a}}(X)$ on the encoded output $X = \underline{\mathtt{CCAAATCT}}$, the following steps are executed:

$\Rightarrow \textsc{Decode}_{\mathbf{a}}(\underline{\mathtt{C}}\mathtt{CAAATCT}) = 0 \times S_{5,7}$
$+ \textsc{Decode}_{\mathbf{a}}(\underline{\mathtt{C}}\mathtt{CAAATCT})$
$\Rightarrow \textsc{Decode}_{\mathbf{a}}(\underline{\mathtt{C}}\mathtt{AAATCT}) = 0 \times S_{5,6}$
$+ \textsc{Decode}_{\mathbf{a}}(\mathtt{AAATCT}),$
$\Rightarrow \textsc{Decode}_{\mathbf{a}}(\underline{\mathtt{AA}}\mathtt{ATCT}) = 2 \times S_{5,5} + 0 \times S_{5,4}$
$+ \textsc{Decode}_{\mathbf{a}}(\mathtt{ATCT})$
$\Rightarrow \textsc{Decode}_{\mathbf{a}}(\underline{\mathtt{ATCT}}) = 16$
$\Rightarrow \textsc{Decode}_{\mathbf{a}}(\mathtt{CCAAATCT}) = 2 \times S_{5,5} + 16 = 550$

5 Experimental Synthesis, Access and Rewrite of DNA Sequences

A total of 27 sequences of length 1000 bps each were designed to encode information retrieved from the Berkeley, Harvard, MIT, Princeton, Stanford, and UIUC Wikipedia page in 2014. Except for sequence #4, which was rejected due to the complexity of its secondary structure, all sequences were synthesized by IDT (Integrated DNA Technologies). In addition, 27 corresponding address primers were synthesized by the same company. The address sequences of the blocks are listed in Section 3.

As a proof of concept, we performed a number of selection and editing experiments. These include selecting individual blocks and rewriting one of its sections, selecting three blocks and rewriting three sections in each, two close to the flanking ends, and one in the middle. The edits involved information about the budget of the institutions at a given year of operation. Detailed information about the original sequences and their rewritten forms is given in the following sections.

Sequence identifier	Number of sequence samples	Length of the edited region (in bps)	Selection accuracy / readout error percentage	Description of editing method
B1-M-gBlock	5	20	5/5/0%	gBlock method
B1-M-PCR	5	20	5/5/0%	OE-PCR method
B2-M-gBlock	5	28	5/5/0%	gBlock method
B2-M-PCR	5	28	5/5/0%	OE-PCR method
B3-M-gBlock	5	$41 + 29$	5/5/0%	gBlock method
B3-M-PCR	5	$41 + 29$	5/5/0%	OE-PCR method

Table S3. Selection, rewriting and sequencing results. Each rewritten 1000 bps sequence was ligated to a linearized pCRTM-Blunt vector using the Zero Blunt PCR Cloning Kit and was transformed into *E. coli.* The *E. coli* strains with correct plasmids were sequenced at ACGT, Inc. Sequencing was performed using two universal primers: M13F_20 (in the reverse direction) and M13R (in the forward direction) to ensure that the entire blocks of 1000 bps are covered.

We denoted the blocks on which we performed selection and editing by B1, B2, and B3. The primers used for performing the edits in the blocks are listed in Table S2. Note that two primers were synthesized for each rewrite, for the forward and reverse direction. In addition, two different editing (mutation) techniques were used, gBlock and Overlap-Extension (OE) PCR; gBlocks are double-stranded genomic fragments that are frequently used as primers, for gene construction or for mediated genome editing. An illustration of editing via gBlocks is shown in Fig. S1. On the other hand, OE-PCR is a variant of PCR used for specific DNA sequence editing via point mutations or splicing. An illustration of the procedure is given in Fig. S1. To demonstrate the plausibility of a cost efficient method for editing, OE-PCR was used with general primers ($\leq$ 60 bps) only. For edits shorter than 40 bps, the mutation sequences were designed as overhangs in primers. Then, the three PCR products were used as templates for the final PCR reaction involving the entire 1000 bps rewrite.

All 27 linear 1000 bps fragments were mixed, and the mixture was used as a template for PCR amplification and selection of the B1, B2 and B3 sequences. The results of selection are shown in Fig S2, where three banks of size 1000 bps are depicted. These banks indicate that sequences of the correct length were isolated. Subsequent sequencing confirmed that the sequences were indeed the user requested B1, B2 and B3 strands. A summary of the experiments performed is provided in Table S3.

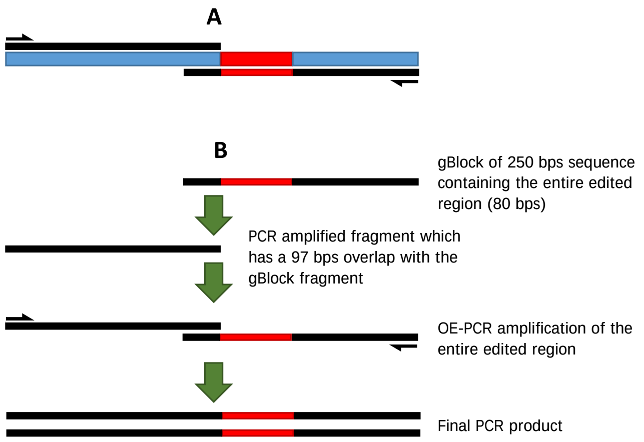

Fig. S1. A) Schematic depiction of the editing method using gBlocks. B) Detailed description of the generation of the mutation. Four sequences (ranging in length from 177 to 588 bps) containing the entire edit region were gBlock synthesized from IDT. The remaining parts of the 1000 bps sequences were PCR amplified. A homology in at least 30 bps between the flanking end sequence of the blocks and the corresponding end of the gBlock fragment was created. By one OE-PCR, the desired edits were generated in a one-pot matter.

5.1 B1 mutation B1-M synthesis

The unedited B1_original (B1) sequence is of the form:

```
AATTACTAAGCGACCTTCTCGGATAGAACGCTTAGTTGGTGCGTTGACAT
GCTCGAACTGATCATCGGTCACTTGCATTCATTATTGATTGTTGAGTTGA
GAAGCGCATTGGTGTCACTCGTTGCTGGGTCATTTTCGGCGAGAGAAACA
GTTCACTGTGGCGTGATGTTTTGAAATGAGGGAGAGTTCTCTTAACTGCA
GTTGGAGTTCAGTATACTCGGGATAGTGTAACAGAGGGAGGCGGATGTGT
GTATTGATGTGAAGTCTTTCACGTGCGGGCTAGGTCGTAATGACGGGTCG
GGAACTATTCATTGGCGCAATAGTGATTTTGATGAATGATGGATAGAACG
CTTAAAGGGAAACTATATAGTTCAAAGCTCGTCGGCGGTGTCGAGGATGT
ATAGGGGTTAATGAATGGTGGAACTTACTTATACTATAGATTGGACTGGT
GGTATGAGAACTTCACTAATTATTGACGTCACAGTTAGTTGTTATGAAGT
GATAATATGAATCGAGCGCAACAGGACTAGTCATTTACTTTTAAGGGAGA
GGAATAGCTAATCTCAAATTTTTTTTATGTGAGTGCACGATCATCACATA
ACATAGGAGGCGATGAGACAGCGACTCAATCTGACTAATTCATTATAGGA
GTTATATGAAGAGTTCGGAACGAAGCTAGCGCTTTCGCACAATGCGAGGG
ATAAGAGCGGGTGCAGAGCGAAGGGTGTGAAATTGATGGTGGATAAGAAC
TTCGCACAGTACTAGCTAGTGGGGAGAGACTTCTATGAATTCGGAGGGAT
ACTTGATATTGATATGGGGGGATGGCGCTATTAAGCGCAGAGCGTAAGTG
CGCTTCAAATCGAACATTGTGTAGCTAAGCAATAGAGAAATGTGGGGATT
GAGCAGTTCGTATCGGTTCGCATGACATACTTGGGAAAATGGCAGCTTGT
TTAAGCTAAACTGGATGAAAGGGAGGAAAAACTTATTGCGACTTCTAAGG
```

where the bases written in red represent the regions we edited.

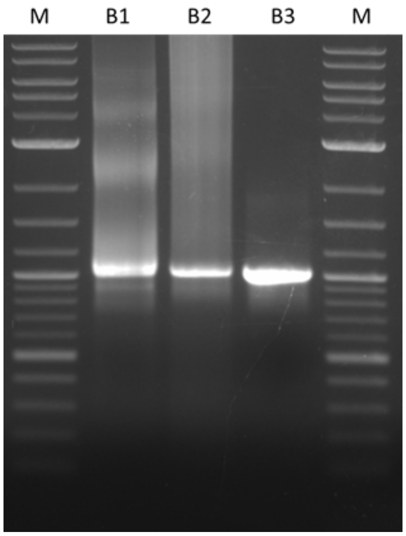

Fig. S2. PCR of 1000 bps sequences-B1, B2, B3 from a mixture of 26 sequences.

The edited B1_mutation (B1_M) sequence reads as:

```
AATTACTAAGCGACCTTCTCGGATAGAACGCTTAGTTGGTGCGTTGACAT
GCTCGAACTGATCATCGGTCACTTGCATTCATTATTGATTGTTGAGTTGA
GAAGCGCATTGGTGTCACTCGTTGCTGGGTCATTTTCGGCGAGAGAAACA
GTTCACTGTGGCGTGATGTTTTGAAATGAGGGAGAGTTCTCTTAACTGCA
GTTGGAGTTCAGTATACTCGGGATAGTGTAACAGAGGGAGGCGGATGTGT
GTATTGATGTGAAGTCTTTCACGTGCGGGCTAGGTCGTAATGACGGGTCG
GGAACTATTCATTGGCGCAATAGTGATTTTGATGAATGATGGATAGAACG
CTTAAAGGGAAACTATATAGTTCAAAGCTCGTCGGCGGTGTCGAGGATGT
ATAGGGGTTAATGAATGGTGGAACTTACTTATACTATAGATTGGACTGGT
GGTATGAGAACTTCACTAATTATTGACGTCACAGTTAGTTGTTATGAAGT
GATAATATGAATCGAGCGCAACAGGACTAGTCATTTACTTTTAAGGGAGA
GGAATAGCTAGCTCTTGAAATATGGGTTATGAGTGCACGATCATCACATA
ACATAGGAGGCGATGAGACAGCGACTCAATCTGACTAATTCATTATAGGA
GTTATATGAAGAGTTCGGAACGAAGCTAGCGCTTTCGCACAATGCGAGGG
ATAAGAGCGGGTGCAGAGCGAAGGGTGTGAAATTGATGGTGGATAAGAAC
TTCGCACAGTACTAGCTAGTGGGGAGAGACTTCTATGAATTCGGAGGGAT
ACTTGATATTGATATGGGGGGATGGCGCTATTAAGCGCAGAGCGTAAGTG
CGCTTCAAATCGAACATTGTGTAGCTAAGCAATAGAGAAATGTGGGGATT
GAGCAGTTCGTATCGGTTCGCATGACATACTTGGGAAAATGGCAGCTTGT
TTAAGCTAAACTGGATGAAAGGGAGGAAAAACTTATTGCGACTTCTAAGG
```

with rewrites listed in red.

5.1.1 The gBlock method

Since a gBlock of length longer than 500 bps was needed, it was more costly to synthesize the gBlock and perform rewriting than to directly re-synthesizing the whole block. Hence, the gBlock method was not used in this case.

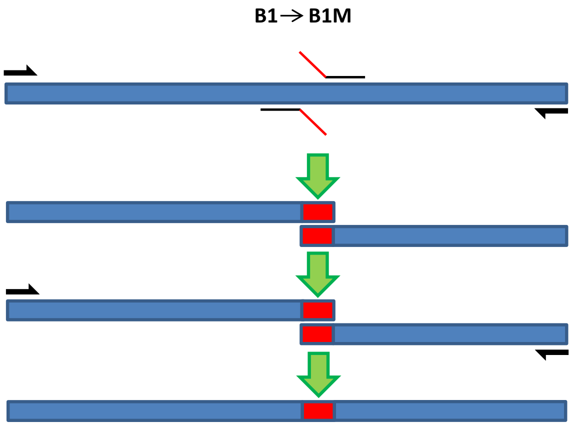

Fig. S3. Illustration of the process of generating the B1 edit/mutation using general primers.

5.1.2 The OE-PCR based method

One pair of primers was designed to PCR amplify the first portion of the sequence B1-M. For the forward direction, the primer was

5'AATTACTAAGCGACCTTCTC3'

while for the reverse direction, the primer was

5'CGTGCACTCATAACCCATATTTCAAGAGCTAGCTATTCCTCTCCCTTAAAAGTAAATGAC3'.

The second part of the sequence was PCR amplified by using the forward direction primer

5'GGGAGAGGAATAGCTAGCTCTTGAAATATGGGTTATGAGTGCACGATCATCACATAAC3'

and reverse direction primer

5'ACTTATTGCGACTTCTAAGG3'.

Both PCR reactions used the sequence B1 as template. Two such PCR products are shown in Fig. S4, indicating that the correct length products were isolated in each reaction.

OE-PCR was performed in a 50 ul reaction volume containing the two aforementioned PCR products without primers for the first 5 cycles and the products with primers (B1 primers in Table S2) for the later 30 cycles. A single bank with correct size of 1000 bps was obtained (see Fig. S4).

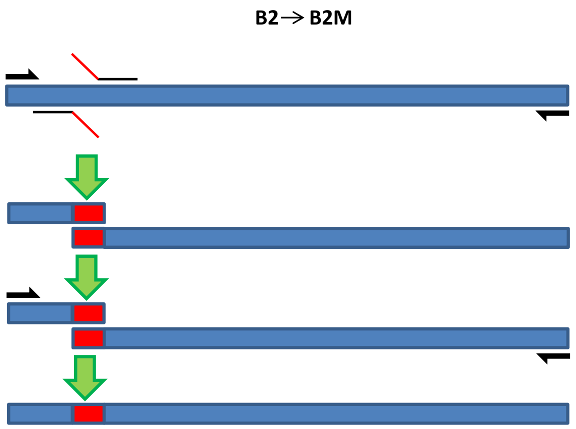

Fig. S4. A schematic depiction of the process of generating the B2 mutation using standard 60 bps primers.

5.2 B2 mutation B2-M synthesis

The unedited B2_original (B2) sequence is of the form:

```
AACCTAACCATCTTCCTCTCGATTTGGAGCAGATTGGTATTATTCTAGTC
GTCGAGACTAGTCAACTGCGCTAGTTTGTGTTCATAAAATAAGAGTATGA
GATACAAGCTGATATGGGAACTTAATTACGAAGCACAGTGTTGCTGCGTG
GACTTGTGAAGTAGGGTGTGAGATAAGAATGATAGCGAACGCAGCGTATG
GCTGAAGTGCTGGGCATATTGTGGTGTGGACATCTCAAAGTCTATGAAGA
TTGGTAATAGGATGGTCTCTCGGGTCTCAAACTTCGTCAGGCAGCATTGT
GCATGCGAGTGATTGAAAGGGAGGGTAAGGGTTATTAATAGAAAAGACTT
ACAGGCGTTGGTATGATTCAAGATCGCAAGAATCGTGTGAGCTTGAGGAC
TAAATAGTTTAAAGAAATAGGAATAGTTGTAATTTAAGGAGCGTGGCACG
GATGGATCAGCGTGTCAACGGAACGCGCATTTGGGAGTTTTATGTTAAGT
GAGCAGACTAAGGTGAAATTCAATAGTCTCTATCGTTCGAGGGTTATTGC
TAGGGGAGACTTTGAGTGAGTGGTAATTTTGAAGCAGTATACGTAACTTT
TTCGATTCTTAGTGGCAGTTACTCTGAATTTTAGTGTGAGCAGAGTGTGA
TAAATAGAGAGATACGAGGTCGACACGGCTGTTGGGGGCACTTAACAGTA
GGGGGTTGATGCTGGCGGACACTAAAGGATTTTTGAAGGGGATTGTTGGC
GACTCACATCTAAGTGGTATTGCGGGCTCTATGAGAATCTGCTCGAGTCA
TCTAGGTTGAGGAAGAGGGGGAGATTCTCGTTAAAGACAGTACATATTTC
GCATACTTCTTAACGTGGAGTATGAATGTCAATGGTGGGAGATATGGGTG
GAGGGATTTCATTCACTGCATATGTACGCTCAGGAGCGCGAACGAATCAT
AAAACTATTGTAATATATTGATAGATAAAGAAACGATCCCCTGACAGAGC
```

The edited B2_mutation (B2_M) sequence is:

AACCTAACCATCTTCCTCTCGATTTGGAGCAGATTGGTATTATTCTAGTC
GTCGAGACTAGTCAACTGCGCTGACGGTTATGGAATTAGGGTTGAGATGG
GATACAAGCTGATATGGGAACTTAATTACGAAGCACAGTGTTGCTGCGTG
GACTTGTGAAGTAGGGTGTGAGATAAGAATGATAGCGAACGCAGCGTATG
GCTGAAGTGCTGGGCATATTGTGGTGTGGACATCTCAAAGTCTATGAAGA
TTGGTAATAGGATGGTCTCTCGGGTCTCAAACTTCGTCAGGCAGCATTGT
GCATGCGAGTGATTGAAAGGGAGGGTAAGGGTTATTAATAGAAAAGACTT
ACAGGCGTTGGTATGATTCAAGATCGCAAGAATCGTGTGAGCTTGAGGAC
TAAATAGTTTAAAGAAATAGGAATAGTTGTAATTTAAGGAGCGTGGCACG
GATGGATCAGCGTGTCAACGGAACGCGCATTTGGGAGTTTTATGTTAAGT
GAGCAGACTAAGGTGAAATTCAATAGTCTCTATCGTTCGAGGGTTATTGC
TAGGGGAGACTTTGAGTGAGTGGTAATTTTGAAGCAGTATACGTAACTTT
TTCGATTCTTAGTGGCAGTTACTCTGAATTTTAGTGTGAGCAGAGTGTGA
TAAATAGAGAGATACGAGGTCGACACGGCTGTTGGGGGCACTTAACAGTA
GGGGGTTGATGCTGGCGGACACTAAAGGATTTTTGAAGGGGATTGTTGGC
GACTCACATCTAAGTGGTATTGCGGGCTCTATGAGAATCTGCTCGAGTCA
TCTAGGTTGAGGAAGAGGGGGAGATTCTCGTTAAAGACAGTACATATTTC
GCATACTTCTTAACGTGGAGTATGAATGTCAATGGTGGGAGATATGGGTG
GAGGGATTTCATTCACTGCATATGTACGCTCAGGAGCGCGAACGAATCAT
AAAACTATTGTAATATATTGATAGATAAAGAAACGATCCCCTGACAGAGC

where, as before, red letters were used to indicate the rewritten region.

5.2.1 The gBlock method

A 177 bps sequence, containing the entire edited region and the B2 string, was gBlock synthesized by IDT. Another part of B2 was PCR amplified using the forward primer

5'GAAGCACAGTGTTGCTGCGTG3'

and reverse primer

5'AAACGATCCCCTGACAGAGC3'

The B2 sequence served as a template. See Fig. S4 for an illustration.

5.2.2 The OE-PCR based method

Over extension PCR (OE-PCR) was performed in a 50 ul reaction volume containing the above 177 bps gBlock product and PCR products without primers for the first 5 cycles and with B2 forward and reverse primers listed in Table S2 for the subsequent 30 cycles.

The PCR product was deposited on a gel substrate and the correct 1000 bps band was obtained as shown in Fig. S5.

One pair of primers was designed to PCR amplify the first part of the sequence B2-M, with forward primer

5'AACCTAACCATCTTCCTCTC3'

and reverse primer

5'CAGCTTGTATCCCATCTCAACCCTAATTCCATAACCGTCAGCGCAGTTGACTAGTCTC3'.

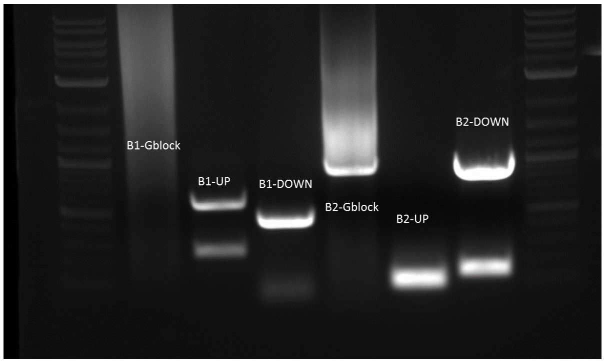

Fig. S5. PCR products of B1 and B2.

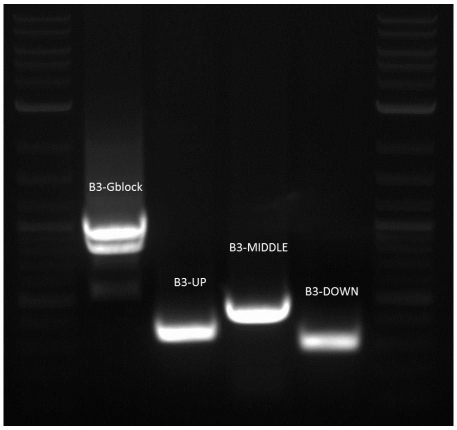

Fig. S6. PCR products of B3.

The second part was PCR amplified by the forward primer

5’CTGCGCTGACGGTTATGGAATTAGGGTTGAGATGGGATACAAGCTGATATGGGAAC3’

and reverse primer

5’AAACGATCCCCTGACAGAGC3’.

Both PCRs used B2 as a template. Two PCR products are shown in Fig. S5.

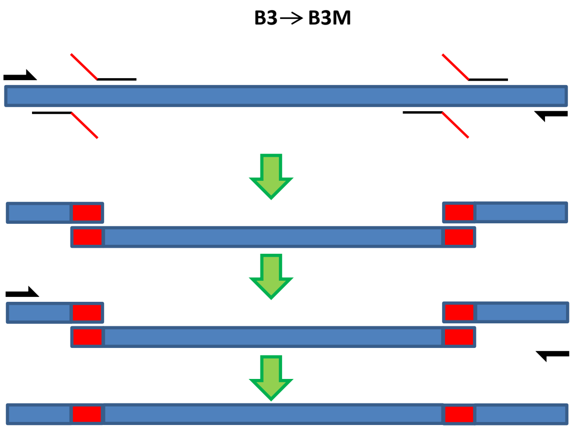

Fig. S7. Scheme for generating the B3 edits using standard 60 bps primers.

5.3 B3 mutation B3-M synthesis

The unedited original B3 sequence equals:

```
ATAATAGGCCTGATGATCTCGATGGATGCGCGTCACTCGAGTGCGGTAGG
CACGTCTCAGGTGATAAGTGATTGTGATTGTAGGTGAAGGGGGTAGAAAT
GATTGAGGAAACTTGTGTACTCGTTACACGTGATAGGGTTTGATCGGCGG
TGGAAAAATTAGGGATGGGGATAAGATTATGGGATCGTTCTCAATAATTG
TTACGATATCGTTGTTACACAGTTGTTACGCTACGACGTCATCGATAAAG
GTGGGTATGTGGGGGTACTATACTCTTGGGGGCGTACAAGAGCGATGGTT
GGTCGGATTGAAATTAAAAGCATTAAGAGGTTAATTTATAGATGCGAGGC
GAAAGATGTGAGCGCAAGTAAAGGAAACGCGAGCAAGTGATTGTTACTAA
TTATATTAGGAGGTGATGAGGAGCGTGGTTATCTTATTGGGCGAGCTGCA
GCGAATTCTAGATTTCTTCGAGTTACAGTCGTAGTGATGTATATAGAGTG
GATGCGCACATTATTACATATATCGTCGAATTGGATTAGACGCAAAGAAA
ATGCGGCATTGTAATGGGTTGTGTAAAATTGAGCGTGGTTATCTTGTCAT
GACATAGTAAAAGTTGCTCAATTGATTGAAGCTCGATTAGGAGAAGTAAT
TTGAAAAAAGGATAGACTAGGACTCAACGAGGAACGGGTATTTGCAACAT
AGTATATGCGGTCTTAATCGGAGGGTAATGTTATTTGTGTGGAAGTCGCT
GCTGGTACTCTGGGCGTTTAGGATGAATCTTCGAAACTAGGCTTTGTCAG
AGATAGTTTGTTGGTAAGAAGAATCAGGAAACGGTAACAGAGAATAAATG
AATTAACGTAGCAAGATTTCGTCTTTCTGGAGATGAGAAGGTGTAGTTGA
GGAGTCGACGTTCTTTACGGAGGTGGGAGATTGGTTTTGGCAGTACTTCG
TTAAATACACTAAAAAAATTTGATAATGTAGAAGAAGAACCAGTAAGCAGC
```

The edited sequence B3_M mutation sequence is:

```
ATAATAGGCCTGATGATCTCGATGGATGCGCGTCACTCGAGTGCGGTAGG
CACGTCTCAGGTGATAAGTGATTGTGATTGTAGGTGAAGGGGGTAGAAAT
GATTGAGGAAACTTGTGTACTCGTTACACGTGATAGGGTTTGATCGGCGG
TGGAAAAAATTAGGGATGGGGATAAGATTATGGGATCGTTCTCAATAATTG
TTACGATATCGTTGTTACACAGTTGTTACGCTACGACGTCATCGATAAAG
GTGGGTATGTGGGGGTACTATACTCTTGGGGGCGTACAAGAGCGATGGTG
TGTACACAGTTCAAGCTTAGATTGAGAGTGAGTAGATGTTGATGCGAGGC
GAAAGATGTGAGCGCAAGTAAAGGAAACGCGAGCAAGTGATTGTTACTAA
TTATATTAGGAGGTGATGAGGAGCGTGGTTATCTTATTGGGCGAGCTGCA
GCGAATTCTAGATTTCTTCGAGTTACAGTCGTAGTGATGTATATAGAGTG
GATGCGCACATTATTACATATATCGTCGAATTGGATTAGACGCAAAGAAA
ATGCGGCATTGTAATGGGTTGTGTAAAATTGAGCGTGGTTATCTTGTCAT
GACATAGTAAAAGTTGCTCAATTGATTGAAGCTCGATTAGGAGAAGTAAT
TTGAAAAAAGGATAGACTAGGACTCAACGAGGAACGGGTATTTGCAACAT
AGTATATGCGGCTTGATCTAGCATTAATGGATTATAGGGGGGAAGTCGCT
GCTGGTACTCTGGGCGTTTAGGATGAATCTTCGAAACTAGGCTTTGTCAG
AGATAGTTTGTTGGTAAGAAGAATCAGGAAACGGTAACAGAGAATAAATG
AATTAACGTAGCAAGATTTCGTCTTTCTGGAGATGAGAAGGTGTAGTTGA
GGAGTCGACGTTCTTTACGGAGGTGGGAGATTGGTTTTGGCAGTACTTCG
TTAAATACACTAAAAAATTTGATAATGTAGAAGAAGAACCAGTAAGCAGC
```

5.3.1 The Gblock method

Two sequences, the 560 bps sequence containing the first mutation region and the second 560 bps sequence containing the second mutation region, were gBlock synthesized by IDT. There was a 60 bps overlap between the two gBlocks.

5.3.2 The OE-PCR method

OE-PCR was performed in a 50 ul reaction volume containing the above two 560 bps gBlock products without primers for the first 5 cycles and additional B3 forward and reverse primers listed in Table S2 for the subsequent 30 cycles. The PCR product was deposited on a gel substrate and the correct 1000 bps band was obtained.

One pair of primers was designed to PCR amplify the first part of the sequence B2-M, using

5'ATAATAGGCCTGATGATCTC3'

in the forward direction and

5'AACATCTACTCACTCTCAATCTAAGCTTGAACTGTGTACACACCATCGCTCTTGTACGCC3'

in the reverse direction.

The second part was PCR amplified in the forward direction by using the primer

5'GTGTACACAGTTCAAGCTTAGATTGAGAGTGAGTAGATGTTGATGCGAGGCGAAAGATGT3'

and in the reverse direction by using the primer

5'GACTTCCCCCCTATAATCCATTAATGCTAGATCAAGCCGCATATACTATGTTGCAAATAC3'.

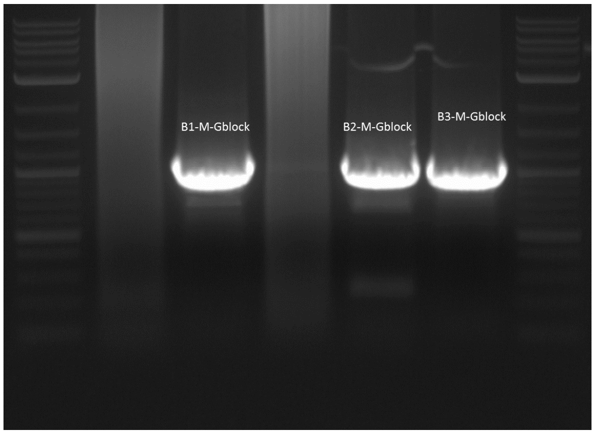

Fig. S8. The generated PCR products of 1000 bps edits from the gBlock method, involving B1-gBlock, B2-gBlock and B3-gBlock.

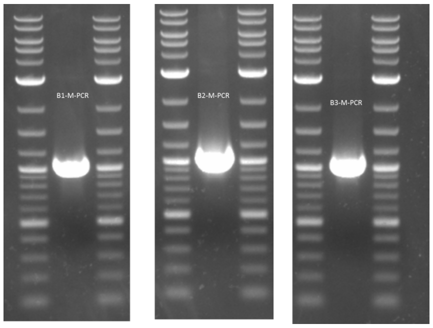

Fig. S9. The generated PCR products of 1000bps sequence editing for the OE-PCR based method, and sequences B1-PCR, B2-PCR and B3-PCR.

The third part was PCR amplified by the forward direction primer

5'GCGGCTTGATCTAGCATTAATGGATTATAGGGGGGAAGTCGCTGCTGGTACTCTG3'

and reverse direction primer

5'AAGAAGAACCAGTAAGCAGC3'.

All three PCRs used the sequence B3 as the template. All three PCR products are shown in Fig. S8.

OE-PCR was performed in a 50 ul reaction volume containing the above three PCR products without primers for the first 5 cycles and with B3 primers listed in Table S2 for the subsequent 30 cycles. A single bank of correct size 1000 bps was obtained (See Fig. S9).

Correctness of the synthesized edited regions was confirmed via DNA Sanger sequencing as follows. The PCR products of the gBlock method and the OE-PCR method were named B1-M-gBlock, B2-M-gBlock, B3-M-gBlock and B1-M-PCR, B2-M-PCR, B3-M-PCR, respectively. All final mutations/edits of PCR products were purified using the QiaGen Gel Purification Kit. The purified 1000 bps edited sequences were blunt-ligated to the vector named pCR^{TM}-Blunt (Fig. S10) using the Zero Blunt PCR Cloning Kit and following the manufacturers' protocol. Five colonies of each PCR-Blunt-mutation were sent to ACTG, Int. Sequencing was performed using two universal primers: M13F_20 (for the reverse direction) and M13R (for the forward direction). Bi-directional sequencing was performed in order to ensure that the entire 1000 bps block was completely covered.

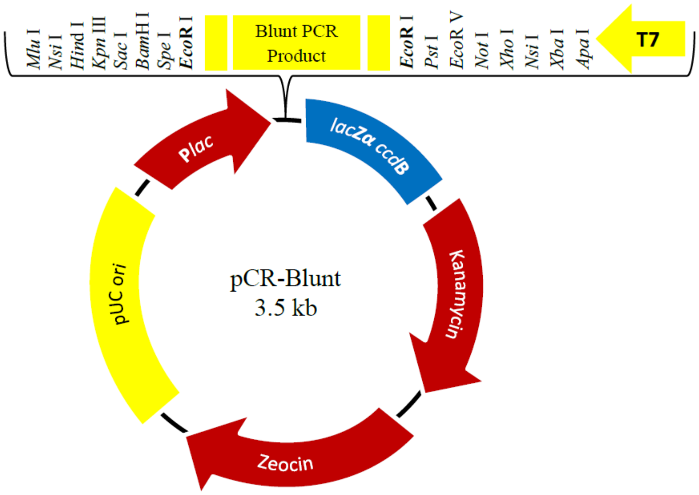

Fig. S10. Map and features of PCR-Blunt vector (Life technologies).

6 Hybrid DNA-Based and Classical Storage

In our small-scale experiments, Sanger sequencing produced two erroneous symbols in one strand which we were able to correct using prefix matching. One possible problem that may arise in large scale DNA-storage systems involving millions of blocks is erroneous sequencing which may not be corrected via prefix matching. In current High Throughput Sequencing technologies, such as Illumina HiSeq or MiSeq, the dominant sources of errors are substitutions. Due to our word grouping scheme, such substitution errors cannot cause catastrophic error propagation, but may nevertheless accumulate as the number of rewrite cycles increases. In this case, prefix matching may not suffice to correct the errors and more sophisticated coding schemes need to be used. Unfortunately, adding additional parity-check symbols into the prefix-encoded data stream may cause problems as the parities may violate the prefix properties and dis-balance the GC content. Furthermore, every time rewriting is performed, the parity-checks will need to be updated, which incurs additional cost for maintaining the system. A simple solution to this problem is a hybrid scheme, in which the bulk of the information is stored in DNA media, while only parity-checks are stored on a classical device, such as flash memory. Given that the current error-rate of short-read sequencing technologies roughly equals 1%, the most suitable codes for performing this type of coding are low-density parity-check codes [2]. These codes offer excellent performance in the presence of a large number of errors and are decodable in linear time.

References

[1] L. J. Guibas and A. M. Odlyzko, "Maximal prefix-synchronized codes," *SIAM Journal on Applied Mathematics*, vol. 35, no. 2, pp. 401–418, 1978.

[2] R. G. Gallager, "Low-density parity-check codes," *Information Theory, IRE Transactions on*, vol. 8, no. 1, pp. 21–28, 1962.
